# A case report of successful identification of ectopic parathyroid adenomas with a sequence of selective parathyroid venous sampling and 4D-computed tomography in a patient with recurrent hyperparathyroidism

**DOI:** 10.1016/j.ijscr.2020.04.021

**Published:** 2020-05-07

**Authors:** Onnicha Suntornlohanakul, Rattana Leelawattana

**Affiliations:** Endocrinology and Metabolism Unit, Division of Internal Medicine, Faculty of Medicine, Prince of Songkla University, Thailand

**Keywords:** Case report, Recurrent primary hyperparathyroidism, Ectopic parathyroid gland, 4D-computed tomography, Selective parathyroid venous sampling

## Abstract

**Introduction:**

Recurrent hyperparathyroidism is difficult to manage due to the difficulty in finding the missing adenoma. Herein we present a case of recurrent hyperparathyroidism from ectopic adenomas which basic investigations failed to locate but were finally localized by a 4DCT following selective venous sampling (SVS) of parathyroid hormone (PTH).

**Presentation of case:**

A young female presented with recurrent hyperparathyroidism. She had severe primary hyperparathyroidism and temporary normocalcemia after first parathyroidectomy. Her hypercalcemia recurred and required second operation. However, the second operation was unsuccessful due to the pre-operation ultrasound, computed tomography (CT) neck, and sestamibi failed to identify the culprit parathyroid adenoma. After the second operation, positron emission tomography (PET), CT neck and sestamibi failed to identify the tumor but a sequence of SVS PTH and four-dimensional computed tomography (4DCT) successfully identified several ectopic adenomas.

**Discussion:**

Ectopic parathyroid tissue is the most common cause of recurrent hyperparathyroidism but precisely locating these ectopic glands is often challenging. Despite modern modalities such as PET scans, the success rate is not impressive. SVS PTH is a good method to regionalize the ectopic source of PTH. With the more specified area, fine-tuning imaging with a 4DCT can identify the specific location of the ectopic parathyroid tissue.

**Conclusion:**

A sequence of SVS PTH followed by 4DCT could identify the exact location of ectopic parathyroid adenomas in a patient when conventional non-invasive imaging studies failed.

## Highlights

• Ectopic parathyroid adenoma is a common cause of recurrent hyperparathyroidism.
• Identification of the ectopic parathyroid adenoma is the key to successful treatment.
• A sequence of PTH venous sampling followed by 4DCT found ectopic parathyroid adenomas when other investigations failed.

## 1 Introduction

Parathyroidectomy is the standard treatment for primary hyperparathyroidism with a good success rate [1]. If hypercalcemia presents within 6 months after the operation, persistent hyperparathyroidism is diagnosed, while recurrent hyperparathyroidism is defined as hypercalcemia which recurs after 6 months post-parathyroidectomy with some period of normocalcemia [2]. These 2 conditions occur in only 2.5–5% of cases [3]. The major risks of recurrent or persistent hyperparathyroidism are multiple gland disease and ectopic parathyroid gland [2,3].

Herein, we present a case of recurrent hyperparathyroidism who failed two parathyroidectomies including a total thyroidectomy and thymectomy in our university hospital, but normal non-invasive imaging failed to locate the ectopic and supranumerical glands, which were finally located by a sequence of selective venous sampling (SVS) of parathyroid hormone (PTH) and four-dimensional computed tomography (4DCT).

This case is reported in accordance with the surgical case report (SCARE) guidelines [4].

## 2 Case presentation

A young female presented with a fragility fracture. She had unremarkable family and other medical history. Her serum calcium and PTH levels were 15.2 mg/dl and 1304 pg/mL, respectively, and she was diagnosed as primary hyperparathyroidism, which fulfilled the indications of parathyroidectomy. An ultrasound revealed a left parathyroid nodule and 99mTC-sestamibi revealed 2 functioning parathyroid nodules, one below the left lobe and the other behind the right lobe of the thyroid gland. A parathyroidectomy was done, removing 2 glands with an isthmectomy and left thyroid lobectomy. Intraoperatively, the main findings were left inferior and right superior parathyroid adenomas. The PTH levels dropped from 1212 at the beginning of the operation to 169.9 at 10 min and 15.84 pg/mL at 24 h post-removal. She also developed hungry bone syndrome after the operation. A histopathological study confirmed a left parathyroid adenoma and right parathyroid gland.

**Fig. 1:**
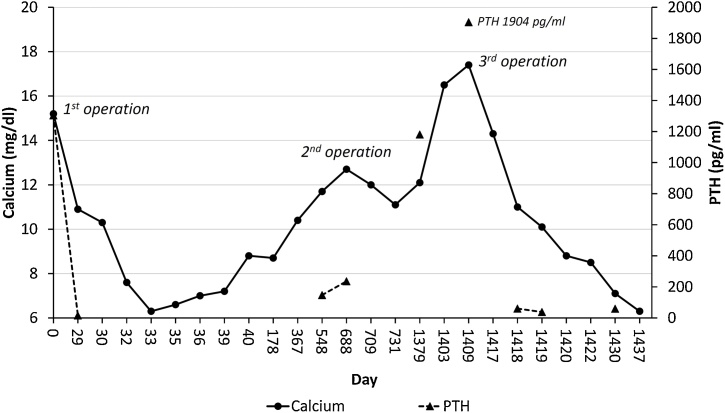
Serum calcium (mg/dl) and PTH levels (pg/mL) during the course of her illness.

**Fig. 2:**
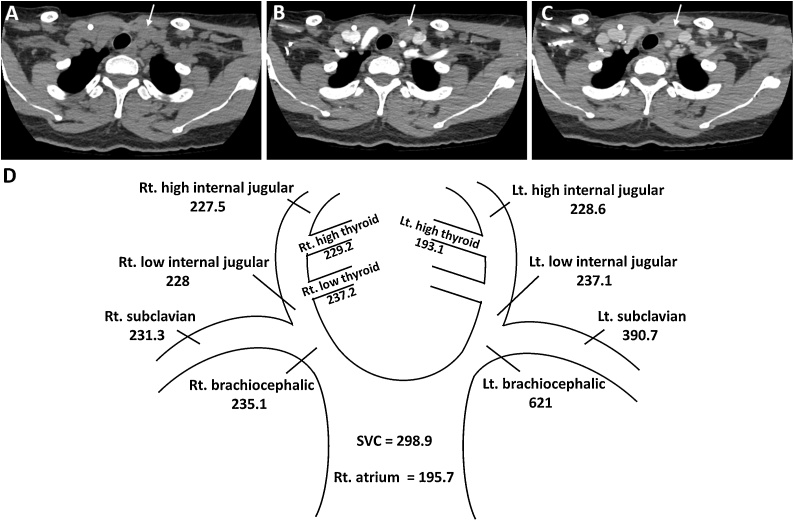
Nonenhanced phase (A), arterial phase (B) and delayed phase (C) axial images of the ectopic parathyroid adenoma (arrow). Results of PTH levels (pg/mL) from selective parathyroid venous sampling in each vein (D).

During follow up, her hungry bone syndrome gradually improved, requiring no calcium and vitamin D and the serum calcium rose to 11.7 mg/dl with high serum PTH level of 147 pg/mL at 18 months after the surgery, indicating recurrent hyperparathyroidism. At that time, an ultrasound and 99mTC-sestamibi did not reveal any abnormalities. A CT of the chest including neck showed 2 suspicious nodules in the left thyroid bed, and a second parathyroidectomy was performed. Intraoperatively, there were possible left and right parathyroid glands which were removed together with the remaining thyroid gland and the thymus. However, the PTH levels did not decline after the operation. Histopathology revealed only a left parathyroid gland without other parathyroid tissues identified. The patient remained hypercalcemic (Fig. 1). An SVS PTH was performed, which indicated that the lesion was in the area of the left brachiocephalic vein (Fig. 2). After biochemical localization, anatomical localization methods (CT chest including neck, 99mTC-sestamibi and positron emission tomography (PET) scan) all failed to identify the precise location of the lesions. She was lost to follow up.

One year after the second operation, she experienced a hypercalcemic crisis which resisted bisphosphonate and cinacalcet and finally required hemodialysis. A third operation was necessary, and a 4DCT of the neck was performed which revealed 2 parathyroid tissue lesions, one at the left lower neck near the left strap muscle just superomedial to the left internal jugular-brachiocephalic vein, and the other superomedial to the first (Fig. 2). This imaging study guided the surgeon to explore the left jugular area and the intraoperative findings revealed one nodule in the left carotid sheath near the ipsilateral strap muscle, and a second nodule inferomedially to the first and a nearby lymph node. All masses were removed and ten minutes later her PTH had dropped from a pre-operative level of 1904 to 234.3 pg/mL. Her serum calcium levels decreased significantly and she developed hungry bone syndrome (Fig. 1). The histopathology reported that all masses including the tissue suspected to be a lymph node were parathyroid hyperplasias.

## 3 Discussion

Recurrent hyperparathyroidism is rare and has no specific guideline for management but there is a suggestion to apply the recommendations for primary hyperparathyroidism as if for a naïve patient with higher thresholds due to the difficulties of re-operation [2]. Our patient originally presented with a fragility fracture with marked hypercalcemia and had surgery. Although the first surgery was successful and she developed hungry bone syndrome, her hypercalcemia recurred. This case demonstrates that hungry bone syndrome occurring after an operation does not reliably predict the long term remission of primary hyperparathyroidism.

Patients with recurrent hyperparathyroidism usually have an underlying cause or causes such as multigland pathology, ectopic parathyroid gland or residual hyperfunctioning parathyroid tissues such as parathyromatosis or parathyroid carcinoma [2,3]. Multigland pathology and ectopic parathyroid gland are the two most common causes of recurrent hyperparathyroidism, therefore pre-operative localization is necessary. The method of localization can be invasive or non-invasive. Most physicians use a stepwise approach from non-invasive to invasive tests. The non-invasive tests are ultrasound, 99mTC-sestamibi, sestamibi-SPECT with CT, CT (preferably 4DCT) and PET with CT. Among conventional imaging studies, 99mTC-sestamibi and ultrasound are the main modalities. The 99mTC-sestamibi provides the best sensitivity (65–67%) compared with CT [5]. The 4DCT and PET with CT are emerging modalities which provide higher sensitivity than ultrasound or 99mTC-sestamibi. Although a 4DCT which shows an avid enhancement of parathyroid tissue in the arterial phase followed by a rapid washout has a sensitivity of 79.3% in detecting parathyroid lesions [6], there are some limitations to the test such as an increased number of contrast-enhanced phases, and it is only suitable for a small region of interest due to the rapid wash-out of the contrast, typically only 70–90 seconds after contrast injection [7]. In studies including patients with multigland disease, the sensitivities of the 4DCT for detecting multigland disease were only 32% [8] and 58% [6] and the sensitivities of 99mTC-sestamibi were 0 [8] and 31% [6], while that of ultrasound was 13.6% [8]. These studies show that non-invasive tests have limitations in their usefulness in localization of hard-to-find lesions.

Invasive testing is required when non-invasive tests give negative or discordant results. The invasive tests are selective arteriography and SVS PTH. SVS PTH is a procedure that measures the PTH in each cervical vein and compares the values between each area. The procedure has been reported to have 75–94.7% sensitivity in detecting the specific area of ectopic parathyroid tissue in recurrent or persistent hyperparathyroidism [9]. However, SVS PTH requires clinical expertise and can be confounded by distortions and variations in the venous drainage. In our case, the SVS PTH guided the surgeon only to the left brachiocephalic area, including the mediastinum and along the carotid artery, which is much too large an area to attempt to find small lesions. With additional information from a 4DCT, we were able to precisely pin point the lesions as being in the left jugular area, which allowed the surgeon to successfully identify the ectopic parathyroid lesions. The combination of 4DCT followed by SVS PTH was studied in a series of recurrent hyperparathyroidism cases and found a sensitivity of 95% compared with 50% in 4DCT alone [10]. Most earlier studies began with non-invasive tests followed by SVS PTH, thus data on the use of SVS PTH followed by 4DCT are limited. Our case describes the approach of using SVS PTH followed by 4DCT in identifying ectopic supranumerical parathyroid glands. When the 4DCT is used in a small area identified by SVS PTH, the rate of successfully localizing ectopic parathyroid tissue should increase. Given our patient’s condition of having supranumerical parathyroid disease and the aggressiveness of disease activity, she will require lifelong monitoring.

## 4 Conclusion

Recurrent hyperparathyroidism is difficult to treat. Precise pre-operative localization is required to detect ectopic parathyroid lesions. We report a patient whose conventional non-invasive imaging studies failed to identify the precise location of the lesions, but the sequence of SVS PTH followed by 4DCT accurately identified the ectopic and supranumerical glands.

## Declaration of Competing Interest

None relates to this report.

## Funding

None.

## Ethical approval

This case report is approved by the Ethics committee of the Faculty of Medicine, Prince of Songkla University.

## Consent

Written informed consent was obtained from the patient for publication of this case report and accompanying images. A copy of the written consent is available for review by the Editor-in-Chief of this journal on request.

## Author contribution

**Onnicha Suntornlohanakul**: Conceptualization, Writing - Original draft preparation, Writing - Review & Editing.

**Ratttana leelawattana**: Conceptualization, Writing- Review & Editing.

## Registration of research studies

Not related.

## Guarantor

Rattana Leelawattana.

## Provenance and peer review

Editorially reviewed, not externally peer-reviewed.
